# Effect of probe characteristics on the subtractive hybridization efficiency of human genomic DNA

**DOI:** 10.1186/1756-0500-3-109

**Published:** 2010-04-20

**Authors:** Marie J Archer, Nina Long, Baochuan Lin

**Affiliations:** 1 US Naval Research Laboratory, Center for Bio/Molecular Science & Engineering, 4555 Overlook Avenue, S W, Washington, DC, 20375, USA; 2 NOVA Research Inc, 1900 Elkin St, Suite 230, Alexandria, VA, 22308, USA

## Abstract

**Background:**

The detection sensitivity of low abundance pathogenic species by polymerase chain reaction (PCR) can be significantly enhanced by removing host nucleic acids. This selective removal can be performed using a magnetic bead-based solid phase with covalently immobilized capture probes. One of the requirements to attain efficient host background nucleic acids subtraction is the capture probe characteristics.

**Findings:**

In this study we investigate how various capture probe characteristics influence the subtraction efficiency. While the primary focus of this report is the impact of probe length, we also studied the impact of probe conformation as well as the amount of capture probe attached to the solid phase. The probes were immobilized on magnetic microbeads functionalized with a phosphorous dendrimer. The subtraction efficiency was assessed by quantitative real time PCR using a single-step capture protocol and genomic DNA as target. Our results indicate that short probes (100 to 200 bp) exhibit the best subtraction efficiency. Additionally, higher subtraction efficiencies with these probes were obtained as the amount of probe immobilized on the solid phase decreased. Under optimal probes condition, our protocol showed a 90 - 95% subtraction efficiency of human genomic DNA.

**Conclusions:**

The characteristics of the capture probe are important for the design of efficient solid phases. The length, conformation and abundance of the probes determine the capture efficiency of the solid phase.

## Findings

The presence of a large excess of non-target nucleic acids (NA), *i.e*. host genomic NA, sometimes is inevitable and can cause false positive or negative results when using molecular diagnostic technologies, such as PCR. The separation of background from low abundance pathogenic targets that coexist in complex matrices is necessary to ensure optimal detection sensitivity and specificity [1-4] and can be done using a solid phase with covalently attached probes. The characteristics of the probe, such as length, conformation and abundance on the solid phase, are of relevance in determining the capture efficiency. Therefore it is necessary to understand how such characteristics affect the capture efficiency in order to design efficient solid phases.

Information on the significance of probe diversification is limited. Most of the results reported to date deal with short single stranded oligonucleotides (20-70 nucleotides (nt)) that may not be suitable for capturing genomic targets [2,5-10]. Mathematical models combined with experimental data, aimed at understanding the hybridization dynamics of DNA to surface-bound probes, have revealed that the kinetics are determined by the amount of probes immobilized on the surface, the length, the concentration, and the size of the target [6,11-15]. Zammateo et al. utilized capture probes of various lengths (56-255 base pair (bp)) immobilized on magnetic microparticles to characterize the capture efficiency of a 435 bp target. They found that the longer probes, which correlated with higher immobilization efficiency, exhibited the best subtraction efficiency and determined the reaction yield. However, these observations were based solely on the size of the probe [16]. These results differ from the observations of other studies which suggest that low probe densities favour hybridization kinetics and that there is a trade-off between the length of the probes and their density on the surface [7,11,13]. These contradictory results highlight the complexity in which solid phase hybridization occurs and further support the need to optimize the capture probe depending on particular application needs.

In this work, we focused on the effects of the length, conformation and the amount of probe on the selective capture of human genomic DNA using a previously developed magnetic bead based solid phase that enables capture of genomic targets in a single step [17-19]. Capture probes of various lengths were synthesized using strand displacement, isothermal amplification and PCR. Our results demonstrated that better capture efficiency was achieved using shorter probes and correlates to the amount of immobilized probes. Furthermore, fragmented human genomic DNA targets (100-5000 bp) can be captured efficiently even with probes as short as 100 to 200 bp. This finding disagrees with the existing literature which suggests that the target should be shorter than 100 bp for efficient solid phase hybridization [11].

## Methods

### Magnetic bead based solid support preparation

Preparation and functionalization of magnetic beads with generation 4.5 phosphorous dendrimer was as described in a previous publication [17] with slight modifications. Briefly, the magnetic beads were prepared in batch mode and the volumes of solvent used were adjusted accordingly [see Additional file 1 for details].

### Preparation of capture probes

Capture probes were prepared by three different methods: Sequenase DNA Polymerase, Klenow fragment, and PCR. The Sequenase probes were prepared in a 30 μl volume containing 40 mM Tris-HCl (pH 7.5), 20 mM MgCl_2_, 50 mM NaCl, 7 μM Primer D [See additional file 1: Supplemental Table S1], 0.67 mM dNTPs, 13 U of Sequenase™ Version 2.0 DNA Polymerase (USB, Cleveland, OH), and 4 μg of COT human DNA with preliminary denaturation at 95°C for 2 min., followed by incubation at 10°C for 5 min., then at 37°C for 60 min. The Klenow probes were prepared in a 50 μl reaction volume containing 10 mM Tris-HCl (pH 7.9), 10 mM MgCl_2_, 50 mM NaCl, 1 mM DTT, 20 μM primer D, 0.2 mM dNTPs, 10 U of Klenow fragment (3' → 5' exo^-^) (NEB, Ipswich, MA) with initial denaturation at 95°C for 5 min. followed by immediate cool down at 4°C, incubation at 37°C for 6 hours and enzyme inactivation at 75°C for 20 min. For reactions using Sequenase DNA polymerase and Klenow fragment, the enzymes and the dNTPs were added after the denaturation step. The PCR probes were prepared in a 50 μl reaction volume with GoTaq^® ^DNA Polymerase (Promega, Madison, WI) using 2 μM Primer NL and 0.8 μM Primer NLN [See additional file 1: Supplemental Table S1] and 400 ng of human genomic DNA (Roche, Indianapolis, IN) as template. The amplification reaction was carried out with preliminary denaturation at 94°C for 2 min. followed by 40 cycles of 94°C for 30 s, 40°C for 30 s, 50°C for 30 s, and 72°C for 2 min., and a final extension step at 72°C for 7 min.

All the products were purified using QIAquick PCR purification kit (Qiagen, Valencia, CA) following the manufacturer's instructions. Prior to immobilization, the PCR probes were denatured at 95°C for 5 min. and cooled to 4°C. Verification of the probes as single strands was performed using a Qubit^® ^fluorometer with the Quant-IT™ ssDNA assay kit (Invitrogen, Carlsbad, CA).

### Immobilization of capture probes

The probe immobilization was performed as described in a previous publication with minor modifications [17]. Briefly, magnetic beads functionalized with a generation 4.5 phosphorous dendrimer were re-suspended in 750 μl of 0.3 M sodium phosphate buffer at pH 9.0 (Na_2_HPO_4_) and sonicated for 10-15 seconds to ensure the beads were dispersed. The beads were incubated overnight at room temperature with three additional buffer changes. Immobilization was performed at room temperature by adding 150 μl of capture probe solution at a concentration of 3, 6, 9, 12 and 15 ng/μl with periodic re-suspension. After immobilization, reduction and blocking for non-specific adsorption was carried out as previously described [17] with slight modifications in the sodium borohydride solution (25 mg instead of 12.5 mg of NaBH_4 _was used) and incubation time (extended to 15 min). The beads were washed with 0.2% SDS, resuspended in 300 μl of stripping buffer (1 × SSC/0.1% SDS) and incubated for 20-24 min. at 95°C with one buffer exchange and re-suspension at the half way point. The beads were then washed with nuclease free water pre-warmed at 95°C for 20-24 min., with re-suspension at the half way point followed by a 30 min. wash in 5 × SCC/0.1% SDS and 0.6 × SCC/0.03% SDS at 55°C with re-suspension at the half way point. Finally, the beads were pooled into 1200 μg aliquots in 0.1% SDS and stored at 4°C after discarding the SDS solution. The immobilization efficiency was determined to be between 95-98% by quantification of the supernatant using a NanoDrop ND-1000 fluorospectrometer (NanoDrop Technologies, Wilmington, DE) as previously described [17]. Magnetic beads with no probes were prepared in an identical manner and used as controls.

### Subtractive hybridization assays

Human genomic DNA was fragmented by McrBC (NEB) supplemented with 0.125 ug/ul of BSA and used as the target. The subtractive hybridization assays were performed as described previously with the following modifications [See additional file 1: Supplemental Figure S1]. The reaction volume was changed to 250 μl and the hybridization buffer (200 μl per sample) contained 3.375 M TMAC/0.11% SDS pre-warmed to 37°C. The reactions were carried out in Thermomixer^® ^(Eppendorf, Westbury, NY) with denaturing at 97°C for 20 min. and annealing at 60°C for 90 min. After incubation, the supernatant was collected and the beads were washed with 100 μl each of 2 × SSC/0.1%SDS and 0.1 × SCC/0.1%SDS. All supernatants were collected and a second magnetic separation was performed to eliminate any bead carryover. The supernatants were ethanol precipitated and resuspended in 10 mM Tris-HCl at pH 8.5.

### Quantitative real-time PCR

Quantitative real-time PCR (qPCR) was performed using the MyiQ™ real-time PCR detection system with iQ SYBR Green Supermix (Bio-Rad Laboratories, Inc., Hercules, CA) according to the manufacturer's recommended protocol. The primers used and PCR conditions are listed in supplemental information [See additional file 1: Supplemental Table S1]. All qPCR results were reported in terms of genome copy concentration per microliter using an external standard curve with known concentration of ACTB.

## Results and discussion

**Figure 1:**
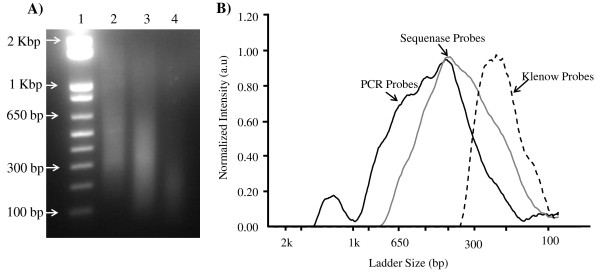
**Capture probe characterization**. (A) Representative image of different capture probes. Lane 1, molecular weight marker; lane 2, PCR probes; lane 3, probes synthesized by strand displacement; lane 4, probes generated by isothermal amplification. The products were run on a 1.2% TAE agarose gel and visualized by ethidium bromide staining. (B) Histogram showing the distribution of the sizes of the capture probes. The corresponding molecular weight marker sizes are indicated in the x-axis.

Three different capture probes for human genomic DNA (hgDNA) were synthesized by isothermal amplification ("Klenow probes"), strand displacement ("Sequenase probes") and polymerase chain reaction ("PCR probes"). Gel electrophoresis of the probes shows a length variation from ~100 - 300 bp for the Klenow probes to ~200 - 600 bp for the Sequenase and ~300 - 800 bp for PCR probes (Figure 1). To compare the subtraction efficiency, the probes were covalently immobilized on magnetic microbeads and subtraction was performed using a previously developed single step protocol [See additional file 1: Supplemental Figure S1]. The supernatant containing the remnant DNA was precipitated and analyzed with qPCR, and the subtraction efficiency for each probe type was compared (Table 1). The results indicated that the Klenow probes showed the best subtraction efficiency with less experimental variation, while the Sequenase probes showed similar subtraction efficiency with higher variations. The PCR probes showed the lowest subtraction efficiency in comparison to the Klenow and Sequenase probes. The results also revealed a general trend that reduction in the amount of probe enhanced the subtraction efficiency. Additional experiments performed at higher probe densities (up to 30 ng/μl) using various buffer systems indicated that the subtraction efficiency decreases as the amount of probe increases (data not shown).

**Table 1:** Subtraction efficiencies of human genomic DNA using three different types of probes

Concentration (ng/μl)	Subtraction efficiency (%) PCR probes	Subtraction efficiency (%) Sequenase probes	Subtraction efficiency (%) Klenow probes
12	66 ± 6	88 ± 3	85 ± 4

6	80 ± 1	77 ± 25	94 ± 5

3	82 ± 6	93 ± 6	95 ± 5

The subtractions were performed in duplicate experiments.

**Figure 2:**
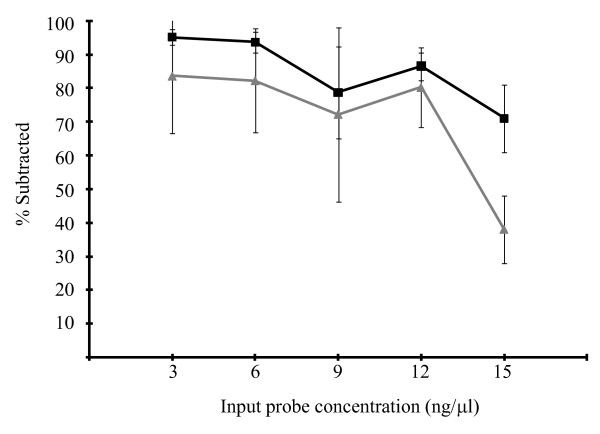
**Comparison of subtraction efficiency between the Klenow and the Sequenase probes**. The Klenow (solid black) and the Sequenase (solid grey) probes were further evaluated for their subtraction efficiency. The Y-axis shows % subtraction, and the x-axis indicates the input probes concentration. These experiments comprised at least 5 subtractions and the qPCR was performed in triplicate. Error bars correspond to the standard deviation of the mean.

Given the low subtraction efficiency of PCR probes, further characterizations were undertaken with only the Klenow and Sequenase probes. Further experiments confirmed the observation that, reduction in the amount of probe enhanced the subtraction efficiency with Klenow probes which exhibited less experimental variations (Figure 2).

These results indicated that the probe length plays a major role in the subtraction efficiency and that the shorter probes provided better subtraction efficiency. This is probably due to the fact that longer probes form secondary structures (hairpin or loop) which are thought to negatively affect the hybridization efficiency in solution and solid phase even with oligonucleotides as short as 60 nt [14,15]. They also represent a more complex scenario since bending induced by electrostatic interactions and intermolecular hybridization can occur and affect hybridization [20]. Our results also indicate that a lower input concentration (3 ng/μl) of shorter probes (~100-200 bp) can be utilized to subtract up to 1000 ng of hgDNA. Larger spacing between the probes reduces steric interference and facilitates diffusion of the targets which is further enhanced by the gradual decrease in temperature and the intermittent mixing. These results contradict the observations by Chan et al. who determined that the rate of hybridization per unit area was equally efficient, regardless of the probe size, if there was proper spacing and as long as the target was between 100 to 160 bp [11]. However, these simulations did not consider secondary structures or targets of variable molecular sizes.

Interestingly, the Sequenase probes contain probes sizes similar to the Klenow probes (100 to 200 bp) (Figure 1), however, the capture efficiency was lower and more variable. This may reflect the fact that longer probes represent the majority within the Sequenase generated probes. In this case the hybridization would be dominated by these longer fragments which exhibit an inherently lower subtraction efficiency than do shorter probes. Even if short probes are present, diffusion of the targets to these sites might be hindered by secondary structure or steric interference. In addition to length, the sequence of the probes is another parameter to consider since it influences the structure of the probes [14,15,21]. However, we have not performed sequencing analysis and we cannot discuss in further detail this particular parameter.

## Conclusions

In conclusion, we have investigated the effect of probe length, conformation and amount on the solid phase capture efficiency of human genomic DNA. In contrast with the published literature, our results indicate that, probes as short as 200 bp can capture between 500-1000 ng of human genomic DNA (100 and 5000 bp). Longer probes (600-1000 bp) exhibited lower subtraction efficiency (~10% difference) with greater variability. Secondary structure and steric interference might be responsible for these differences. In all cases, a lower amount of immobilized probe on the magnetic beads correlated with an enhanced performance. The results presented here are of relevance for the design of efficient solid phases for the selective capture of genomic targets.

## List of Abbreviations

NA: nucleic acids; SDS: sodium dodecyl sulfate; SSC: standard sodium citrate; PCR: Polymerase chain reaction; TMAC: tetramethylammonium chloride.

## Competing interests

There are two pending patent applications, one for preparation and functionalized of magnetic beads and one for genomic DNA subtraction protocol, that are related to this article. Both MJA, and BL are listed as inventors of these two patent applications.

## Authors' contributions

MJA participated in the concept development, characterization and fabrication the solid phase, and drafting the manuscript. NL performed the probes synthesis and subtraction experiments. BL participated in the concept development, performing quantitative real time PCR analysis, and drafting the manuscript.

## Supplementary Material

Additional file 1 **Supplemental material to the methods**. Details on the magnetic bead bases solid support preparation, hybridization capture assays (Figure S1) and primer sequences and PCR conditions (Table S1). Click here for file
