# Supplementary material for: Effect of probe characteristics on the subtractive hybridization efficiency of human genomic DNA
Source: BMC Res Notes. 2010 Apr 20;3:109. doi: 10.1186/1756-0500-3-109 (PMC2862039; doi:10.1186/1756-0500-3-109)
Supplement: Additional file 1 — Supplemental material to the methods. Details on the magnetic bead bases solid support preparation, hybridization capture assays (Figure S1) and primer sequences and PCR conditions (Table S1). [file 1756-0500-3-109-S1.DOC]

# Supplemental Materials and Methods

**Magnetic bead based solid support preparation**

Magnetic silica particles (1 μm) with primary amine functionalities (NH_2_) were purchased from Chemicell GmbH (Berlin, Germany), and functionalized with a generation 4.5 phosphorous dendrimer (Sigma-Aldrich, St. Lois, MO) in batch mode. For this purpose, aliquots of 12 mg of beads were dispensed into six 2-ml polypropylene tubes and washed once with 250 μl of DI water. Since the dendrimer immobilization occurs in dichloromethane, which is insoluble in water, it is necessary to perform a solvent exchange. The beads were washed twice with 400 μl of 50% ethanol and with 800 μl of 100% ethanol, then with 1 ml of dichloromethane. After washing, the magnetic beads were resuspended in 1 ml of dichloromethane and incubated for 12 hours at room temperature with shaking at 90 rpm using an orbital shaker. After incubation, the beads were washed with 1 ml of ethanol, 1 ml of dichloromethane and again with 1 ml of ethanol before transferring them to fresh tubes. The ethanol was removed and 1.5 ml of 2% (w/v) solution of a generation 4.5 dendrimer in dichloromethane was added to each tube, and the beads were incubated for 22 hours at room temperature on an orbital shaker set at 90 rpm. After incubation, the beads were washed four times with dichloromethane, three times with 100% ethanol and resupended in 1 ml of ethanol. The beads were aliquoted into 1.5 ml polypropylene tubes (~ 600 μg of beads), the ethanol was removed, and dried in a dry block heater at 55 ^o^C for 10-15 minutes and stored at room temperature until ready to use.

**Hybridization capture assays**

The hybridization capture assay consists of various steps with intermittent shaking and it is schematically shown in Figure S1.

**Supplemental figures.**

**Figure S1- Steps performed in the subtractive hybridization assay.** This figure depicts the processes for performing subtraction hybridization assay. The assay consists of two major steps: denature and anneal which are performed with intermittent shaking for variable periods of time. The collected supernatants form the capture and the post-capture washes are collected and precipitated for quantification and real time PCR analysis.


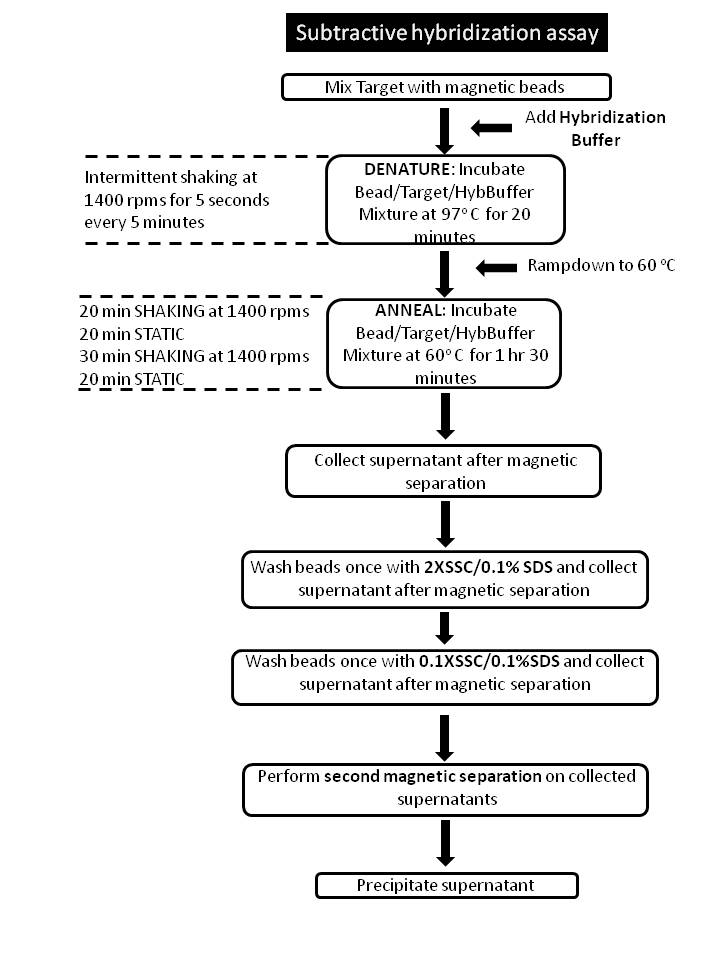


**Supplemental tables.**

**Table S1 -**Primers used for capture probe synthesis and real-time PCR

| **Name** | **Sequence (5’ → 3’)** | **PCR condition** |
| --- | --- | --- |
| Primer D | NH_2-_GTTTCCCAGTAGGTCTCNNNNNNNN |  |
| Primer NL | NH_2-_CGATACGACGGGCGATCTAGC |  |
| Primer NLN | NH_2-_CGATACGACGGGCGATCTAGCNNNNNNN |  |
| hACTB-OF321 | AGAAAATCTGGCACCACACC | (40 cycles) 94 ^o^C, 20s, 54 ^o^C, 20s, 72 ^o^C, 30s. |
| hACTB-OR805 | GTCAGGCAGCTCGTAGCTCT |
| hACTB-F321 | AGAAAATCTGGCACCACACC | (40 cycles) 94 ^o^C, 10s, 50 ^o^C, 10s, 72 ^o^C, 20s. |
| hACTB-R508 | AGAGGCGTACAGGGATAGCA |

Note: hACTB-OF321/OR805 primer pair was used to generate PCR products for standard curve; hACTB-F321/R508 was used for quantitative real-time PCR.
